# Asymmetric gating of a human hetero-pentameric glycine receptor

**DOI:** 10.21203/rs.3.rs-2386831/v1

**Published:** 2023-01-10

**Authors:** Xiaofen Liu, Weiwei Wang

**Affiliations:** University of Texas Southwestern Medical Center; University of Texas Southwestern Medical Center

## Abstract

Hetero-pentameric Cys-loop receptors constitute a major type of neurotransmitter receptors that enable signal transmission and processing in the nervous system. Despite intense investigations in their working mechanism and pharmaceutical potentials, how neurotransmitters activate these receptors remain unclear due to the lack of high-resolution structural information in the activated open state. Here we report near-atomic resolution structures in all principle functional states of the human α1β GlyR, which is a major Cys-loop receptor that mediates inhibitory neurotransmission in the central nervous system of adults. Glycine binding induced cooperative and symmetric structural rearrangements in the neurotransmitter-binding extracellular domain, but asymmetrical pore dilation in the transmembrane domain. Symmetric response in the extracellular domain is consistent with electrophysiological data showing similar contribution to activation from all the α1 and β subunits. A set of functionally essential but differentially charged amino-acid residues in the transmembrane domain of the α1 and β subunits explains asymmetric activation. These findings point to a gating mechanism that is distinct from homomeric receptors but more compatible with heteromeric GlyRs being clustered at synapses through β subunit–scaffolding protein interactions. Such mechanism provides foundation for understanding how gating of the Cys-loop receptor members diverge to accommodate specific physiological environment.

## Introduction

Mammalian Cys-loop receptor superfamily encompasses ionotropic neurotransmitter receptors including cation-selective type 3 serotonin receptors (5-HT3) and nicotinic acetylcholine receptors (nAChR), as well as anion-selective glycine receptors (GlyR) and GABA(A) receptors. These receptors underly fast synaptic and extra-synaptic neurotransmission throughout the nervous systems. They also mediate communication between the nervous and locomotive systems^1^. A myriad of neurological disorders including Alzheimer’s disease, schizophrenia, epilepsy and autism^1–6^, as well as locomotive problems such as hyperekplexia and myasthenic syndrome^7–9^ are caused by or associated with their dysfunction.

Despite decades of intense research, (near) atomic resolution structures in the activated open state has only been characterized for the homomeric receptors, including the homomeric glycine receptors that are found almost solely in embryos^10–13^, and the homomeric α7 nAChR and 5-HT3A receptors that represent only one of the many subunit compositions found in tissues^14,15^. All the homo-pentameric structures point to an open mechanism through symmetric expansion of the ion conduction pathway^10,11,16,17^ (with the exception of 5-HT3A where asymmetric dilation was recently reported^15^). For the predominant heteromeric receptors, although the variations in sequence and chemical property in the constituting subunits may lead to asymmetric gating, cooperativity of neurotransmitters in receptor activation suggests otherwise. Unfortunately, structures of heteromeric receptors have only been captured in either the resting/inhibited closed state, or the desensitized state^18–20^, leaving this conundrum unresolved and details of open state unclear.

GlyRs are major Cys-loop receptors that mediate fast inhibitory neurotransmission in the spinal cord and brain^13,21,22^. Dysfunction of GlyR causes the congenital disorder hyperekplexia^7,8,23–25^. It is a therapeutic target in neuropathic pain^8,26–28^, and related to autism and other neurological disorders^4,13,22,29^. The widely studied stereotypical homomeric GlyRs contain only the α subunits and practically do not exist in adult animals. On contrary, the heteromeric GlyRs containing both the α and the β subunit are the found throughout the central nervous system^13^. Structural information of heteromeric GlyRs has only become available very recently, revealing an unexpected 4:1 α2:β subunit stoichiometry^18,30^. Unfortunately, none of the structures reported was in the activated open state, although a “semi-open” state in one study^30^ had its ion conduction pore asymmetrically expanded to a size just shy of the predicted range of open GlyR pores^22,30,31^.

In this study, we resolved near-atomic resolution structures of the α1β GlyR with native function in all principle functional states throughout its gating cycle: apo (closed), glycine-bound desensitized and glycine-bound open. Combining with electrophysiology and mutagenesis experiments, we show that glycine binding to the orthosteric site results in highly cooperative and pseudo-symmetrical conformational changes in the extracellular domains (ECD), explaining the cooperativity of glycine in activation of GlyRs. However, due to the distinct characteristics of the α and β subunit transmembrane domains (TMD), symmetric activation in ECD resulted in asymmetrical structural rearrangements in the TMD, leading to asymmetrical open conformations that is distinct from homomeric GlyRs. Such differential responses in ECD and TMD reconciliates the cooperativity in glycine activation and differences in subunit properties in an asymmetric gating mechanism. This mechanism ensures that the clustering of GlyRs at synapses through its β subunit does not cause aberrant channel activation independent of glycine. In addition, it explains how mutations of a charged amino-acid residue pointing away from the ion conduction pore diminishes GlyR conductance and cause hyperekplexia.

## Results

### Overall architecture of the human α1β GlyR

**Figure 1:**
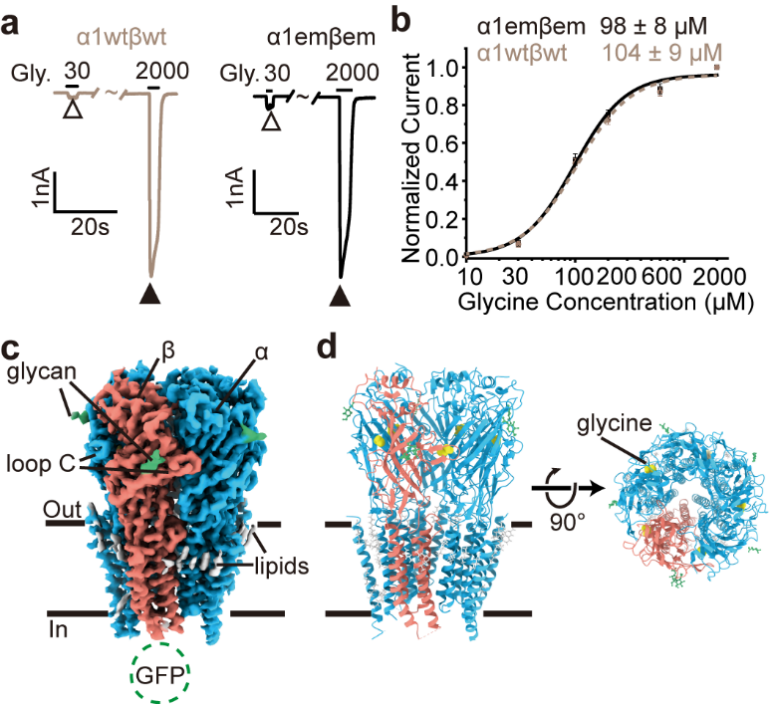
Dose response of glycine and overall structure of the 1β GlyR (a) Typical glycine response of 1wtβwt and 1emβem at 30 μM and 2 mM. (b) Dose response with Hill fits (lines). Data are represented as mean ± SEM (n=7 cells). (c) Side view of cryo-EM map of 1β GlyR in complex with glycine. (d) Side (left) and top-down (right) view of the atomic models. subunits, β subunit, N-glycan, lipids, and glycine are respectively colored in sky bule, salmon, green, gray, and yellow.

To understand gating mechanisms of the human α1β GlyR without complications from function-modifying antibodies and limitations in purification from native tissues, we generated constructs of the α1 and β subunits (α1em and βem, respectively) that form α1emβem GlyR and exhibit indistinguishable function as wild type. α1em contained a partial truncation of the unstructured M3-M4 loop (Supplementary Fig. 1a). βem has been described previously^32^, which contained substitution of GFP for part of the unstructured M3-M4 loop that does not bind with synaptic scaffolds (Supplementary Fig. 1b). These constructs greatly improved biochemical properties of heteromeric GlyR (Supplementary Fig. 1c) during purification. In electrophysiological recordings, α1emβem GlyR and wild-type α1wtβwt GlyR shared the same apparent glycine EC_50_ ~ 100 μM, Hill coefficient (n ~ 1.7) and similar current magnitude across cells (Fig. 1a, b; Supplementary Fig. 1d, e). Apparently, α1emβem GlyR well recapitulates the functional properties of wild-type GlyR.

**Figure 2:**
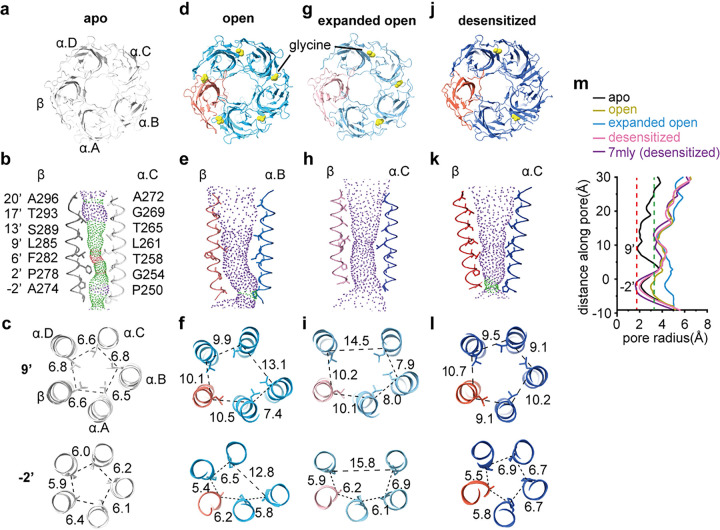
1β GlyR structures in major functional states. (a, d, g and j) Top-down views in the apo (a), open (d), expanded-open (g) and desensitized (j) states. 1 subunits and β subunits are colored in different shades of blue and orange, as indicated, except for in the apo state where β is in gray and 1 is white. (b, e, h and k) Ion permeation pathways in the apo (b), open (e), expanded open (h), and desensitized (k) states. M2 helices are shown as cartoon and the side chains of pore-lining residues as sticks. Purple, green, red spheres define radii of > 3.3 Å, 1.8–3.3 Å, and < 1.8 Å, respectively. (c, f, i and l) Cross-sections of M2 helices at residues 9’ (top) and −2’ (bottom) in the apo (c), open (f), expanded open (i) and desensitized (l) states, with distances between neighboring Cα shown in Å. (m) Plot of pore radii calculated by the HOLE program for the apo (black), open (yellow), expanded open (sky blue), desensitized (hot pink) states, as well as that of porcine desensitized state (PDB ID: 7mly, purple).

Through single-particle cryo-EM analysis, we resolved structures of α1emβem GlyR both in the absence and in the presence of glycine. The density arising from GFP fusion on the βem subunit served as fiducial marker to differentiate the β subunit from the structurally similar α subunits^30^ (Fig. 1c; Fig. 2a, b, i, j), without the use of available subunit-specific antibodies that alter function^18^. The overall resolution ranged from 3.6 – 4.1 Å, with local resolutions extending well beyond 3.0 Å for many regions of interest ( Fig. 2d–f, l–n). These density maps allowed for unambiguous model building for most part of the protein (Fig. 2d; Supplementary Fig. 3; Supplementary Table 1). In all the structures determined, either with or without glycine, 4 α1 subunits and 1 β subunit were identified (Fig. 1c, d), consistent with the recent discovery of 4:1 α2:β stoichiometry of human α2β GlyR and heteromeric GlyR purified from porcine tissue^30^. These structures suggest that 4:1 α1:β stoichiometry is an intrinsic property of α1β GlyR, not dependent on the presence of other GlyR subunits (such as α2) or unidentified factors/chaperons in native tissues^18^.

### α1β GlyR in the closed, open and desensitized states

In the absence of glycine (apo state), a single conformation was resolved that corresponds to the closed states ((Fig. 2a–c). All the orthosteric sites are empty (Fig. 2a). The activation gate^11^ at 9’ is tightly constricted with the 9’ Leu sidechains pointing toward the pore (Fig. 2b, c). The minimal pore radius is ~1.8 Å, too narrow for Cl^−^ to pass (Fig. 2m black). This conformation resembles that of homomeric GlyR in the closed state^11^, as well as the pseudo-symmetrical α2β-strychnine complex in the closed state^30^.

In the presence of glycine, two structures were resolved with widened pores allowing Cl^−^ to pass through (Fig. 2d–i). In one structure, clear glycine densities are found in all 5 orthosteric sites (Fig. 2d). Compared to the apo state, 9’ Leu side chain flipped away from the pore, combined with outward movement of the M2 helix, resulted in an open activation gate^11^. The −2’ position dilated in an apparently asymmetrical manner (Fig. 2 e, f). The minimal radius along the pore is ~ 2.9 Å (Fig. 2m yellow), sufficient to allow partially hydrated Cl^−^ through, and within expected range of physiologically pore sizes of open GlyR^12,31^. This structure should represent a conformation of α1β in the open state.

In the other structure, the orthosteric sites at all 3 α/α interfaces have clear glycine densities, while sites at α(+)/β(−) and β(+)/α(−) interfaces are empty (Fig. 2g, Supplementary Fig. 3). Nonetheless, the pore also dilated in an asymmetrical manner, but to a larger extent, resulting in a minimal radius of 3.5 Å (Fig. 2h, i and m cyan), which is too large for a physiologically open GlyR. Over-widened pore is not likely a result of truncation in the unstructured M3-M4 loops considering the functional equivalence of α1emβem GlyR with wild type (Fig. 1; Supplementary Fig. 1). In addition, a similar “expanded open” conformation has been recently reported of full-length homomeric GlyR^12^.

A third structure was resolved in the α1emβem GlyR – glycine complex that corresponds to the desensitized state. 2 out of 5 orthosteric sites are occupied with glycine (Fig. 2j; Supplementary Fig. 3). The activation gate around 9’ Leu is open. The desensitization gate is constricted to a radius of ~ 2 Å ((Fig. 2k, l, m pink), rendering the channel non-conductive. The pore shape in this structure is similar to that of α2β GlyR^30^, and GlyR purified from porcine tissue in the desensitized state^18^ (Fig. 2m pink and purple), retaining mostly 5-fold pseudo-symmetric shape in the TMD.

The above structures in all the major functional states depict a gating mechanism that has resemblance but is also very different from homomeric GlyRs. Homomeric GlyRs maintain the 5-fold symmetric structure throughout the gating cycle, with all 5 orthosteric pockets in an identical ligand-binding state^10–12,17,33,34^. α1β GlyR changes its symmetry during gating and exhibit varying glycine occupancy in the 5 orthosteric sites. This provides a unique opportunity to understand how asymmetrical gating works in a multimeric ligand-gated ion channel containing non-equivalent subunits.

### Symmetric glycine-induced response in the ECD

**Figure 3:**
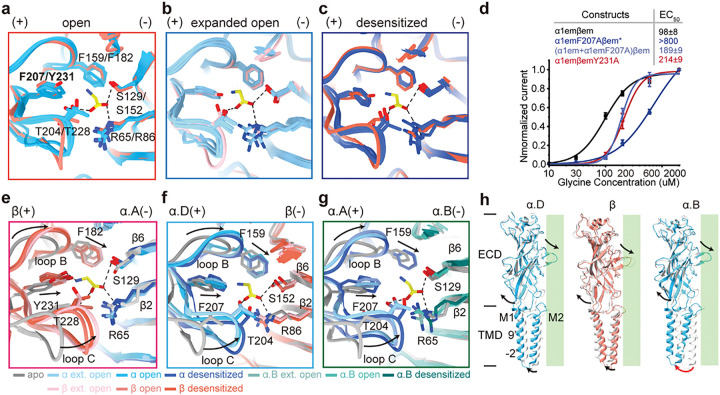
Propagation of glycine-induced structural changes. **(**a, b and c) Overlay of all 5 glycine binding pockets in each of the open (a), expanded open (b) and desensitized (c) states, with (+) and (−) sides indicated. The key amino acids for glycine binding are shown. (d) Dose response of glycine for (+) ( 1emF207Aβem) and β(+) side mutants ( 1emY231Aβem). Data are represented as mean ± SEM (n = 6–9 cells). EC_50_ from Hill fits are listed. (e, f and g) The orthosteric pocket changes from apo state to open, expanded open and desensitized states, at .D(+)β(−) (e), β(+) .A(−) (f) and .A(+) .B(−) (g) subunit interfaces. The key amino acids are indicated. (h) Superimposition of individual subunits in the apo ( : white, β: grey) and open states ( : blue, β: salmon). Rotational motion of ECD propagates to TMD in different ways. Arrows indicate the direction of motion. Green rectangles represent the pore axis.

Glycine binding induced similar conformational rearrangements across all 5 subunits. Binding of agonists is known to induce a compact conformation in the orthosteric site^10,11,18,35,36^. In our apo structure (Fig. 2a), all 5 binding pockets are empty and in the same apo conformation (Supplementary Fig. 4a). In the open state structure, glycine is found in all 5 pockets, resulting in the same compact conformation across all pockets (Fig. 3a, Supplementary Fig. 4b). However, in the expanded-open and desensitized states, orthosteric pockets are only partially occupied (3 and 2 out of 5, respectively) (Fig. 2g, i). Nonetheless, the same compact conformation was observed across all 5 pockets, irrespective of whether glycine is bound (Fig. 3b, c). In addition, ECDs from all 5 subunits showed similar rocking motions that propagate to TMD during channel activation (Fig. 3h). Such symmetric change in the ECD upon glycine binding suggests cooperativity among orthesteric pockets, which is indicated by Hill slopes ranging from ~ 2–5 in our and previously reported glycine-dose response curves (Fig. 1 and Supplementary Fig. 5) ^22,30,37,38^.

All orthosteric pockets contribute similarly to the gating of α1β GlyR. This is indicated by the same conformation across all binding pockets irrespective of subunit type. To further test this, we mutated one amino acid residue (α:F207 or β:Y231, see Fig. 3a) that is important for glycine binding, and performed glycine titration (Fig. 3d). Mutating α:F207 resulted dramatic decrease in apparent glycine affinity, to the point where only a lower limit of EC_50_ ~ 0.8 mM can be estimated (Fig. 3d, blue), while mutating the homologous β:Y231 only resulted in ~ 2 fold increase in EC_50_ (Fig. 3d red). This is because there is only 1 β subunit but 4 α subunits in each GlyR. Consistent with this, mutating a subset of α:F207 resulted in a similar effect as the β:Y231 mutation (Fig. 3d, light blue). This phenomenon is consistent across extensive mutagenesis experiments in glycine binding pockets (Supplementary Fig. 5). The equivalence in response to glycine binding of all subunits is expected considering the cooperativity among these sites, which ensured a pseudo-5-fold symmetric structure in the extracellular domain throughout the gating cycle (Supplementary Fig. 6a, c, e, g).

Interestingly, glycine-induced structural changes in the ECD are similar across the open, expanded-open and desensitized states (Fig. 3e–g). Orthosteric pockets at the β(+)α(−) (Fig. 2e), α(−)β(+) (Fig. 2f), and α(+)α(−) (Fig. 2g) subunit interfaces showed similar contraction upon glycine binding, which in turn induced a rotational motion of the ECD (Fig. 3h) ^10,11^. ECD rotation moved the directly connected extracellular end of M1 helix away from the conduction pore, resulting in an outward symmetric expansion at the ECD-TMD interface (Supplementary Fig. 6i, j). 5-fold pseudosymmetry is maintained through all functional states in ECD and the extracellular end of TMD. However, the conformations of TMD near the intracellular side become more distinctive between different subunits (Fig. 3h). Apparently, the TMD of heteromeric GlyR converts the same symmetric input from ECD into different, sometimes structurally asymmetrical, functional states.

### Asymmetrical gating in the α1β GlyR TMD

**Figure 4:**
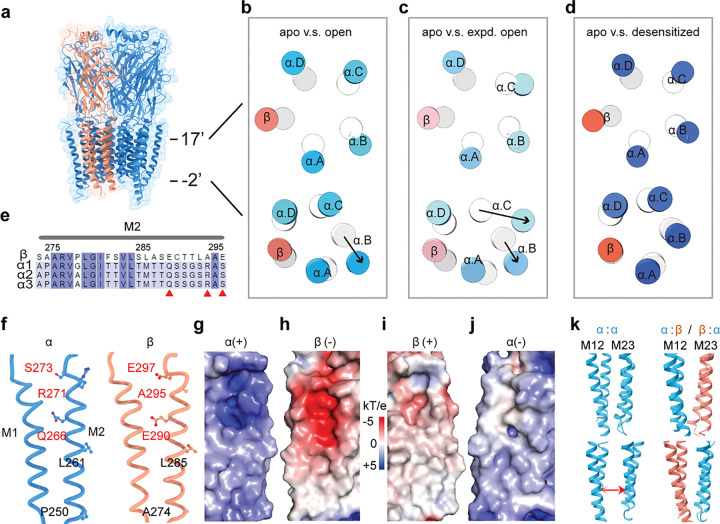
Asymmetric conformational changes in TMD. (a)Side view of the 1β GlyR. β: dark salmon; : blue. The position of 17’ and −2’ are labeled. (b, c and d) Cross sections at 17’ and −2’ of M2 of the apo (gray/white) and open/expanded open/desensitized states. 1 subunits and β subunits are colored in different shades of blue and orange. Black arrows indicate the movement direction. (e) Sequence alignment of M2 helices among human GlyR β, GlyR 1, GlyR 2, GlyR 3. (f) The position of key amino acids in the helix. The corresponding amino acids are marked in panel E. (g, h, i and j) TMD surface colored according to electrostatic potentials (calculated using APBS tools) at (+) (g), β (−) (h), β(+) (i) and (−) (j) interfaces. (k) The cartoon representation of the TMD interfaces between (blue) - β (salmon), and - subunits in the open state. Red arrow indicates increased distance between adjacent subunits.

α1β GlyR in the apo and desensitized states, but not in the open states, retained 5-fold pseudo-symmetry. As described above, the ECD remains largely symmetric throughout the gating cycle. Such symmetry is retained in the TMD in the apo and desensitized states (Fig.4a, d; see also Fig. 2 and Supplement Fig. 6). In the open state, the extracellular end of all 5 TMDs moved radially away from the conduction pore, resulting in a symmetric expansion similar to the desensitized state (the pore-lining M2 helices are shown in Fig. 4a, b, d top panel). However, the expansion became asymmetrical near the intracellular end – in addition to small radial expansion, one of the α1 subunits (chain B) moved away in both radial and tangential directions, creating a wider spacing from one adjacent α subunit (chain C) (Fig. 4b lower panel). This movement resulted in an asymmetrically widened desensitization gate that allows the conduction of Cl^−^. The expanded-open state showed resembling, but more extended structural rearrangements near the desensitization gate – two of the α1 subunits (chain B and C) moved in both radial and tangential directions, creating an asymmetrical wide-open pore. Intriguingly, none of the α1 subunit adjacent to the β subunit experienced such large movements, which we believe is resulting from amino-acid residues unique to each subunit types, as discussed below.

Differentially charged amino acid residues in the α1 and the β M2 helices promote asymmetrical gating. The pore-lining M2 helix is one of the least conserved regions between the α and β subunits in amino acid sequences (Fig. 4e), harboring disease-causing mutagenetic sites and resulting in distinctive functional features including glycine dose-response, single-channel conductance and picrotoxin sensitivity in homo- and hetero-meric GlyRs^13,22,30,37,39^. A set of unique amino acid residues resulted in opposite electrostatic fields at subunit interfaces in TMD of α1 and β subunits (Fig. 4f–j). The combination of two neutral and one positively charge residues, α1:S273/R271/Q266, makes the TMD of α1 positively charged at both the (+) (Fig. 4g) and (−) (Fig. 4j) sides. The β subunit has two negatively charged residues and one hydrophobic residues at corresponding positions; β:E297/A295/ E290, which renders negative potentials at both (+) and (−) sides (Fig. 4h, i). Opposite potentials promote the interaction between the α1 and β subunit TMDs (Fig. 4g binds with h, i binds with j), especially in the hydrophobic environment inside the membrane, leading to a stable α1-β-α1 assembly throughout the gating cycle (Fig. 4b–d, Fig. 2c–l). However, the same positive potential contributes to repulsion between α1-α1 interfaces, increasing the likelihood of widening in α1-α1 interfaces. This explains the widening of some α1-α1 but not the α1-β-α1 interfaces in both the open (Fig. 4k) and expanded-open states.

## Discussion

**Figure 5:**
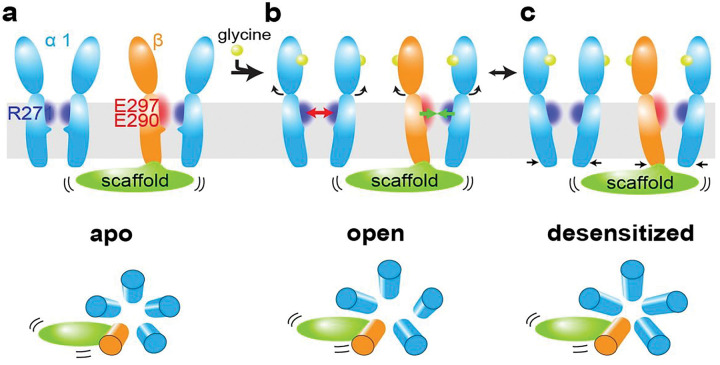
Proposed gating mechanism of the 〿1β GlyR. Adjacent (blue) and β (orange) subunits are shown with respectively charged amino acid residues and surrounding electrostatics (positive: blue; negative: red). Post-synaptic scaffold is shown as green oval. (a) In the apo state, heteromeric 1β GlyR is pseudo-symmetrical with a closed pore. (b) Upon glycine (yellow spheres) binding, conformational changes in ECD cause the widening of the extracellular end of the TMD. Electrostatic repulsion between adjacent subunits makes them easier to separate (red arrow). On contrary, the opposition electrostatics of and β subunits ensures that they stay close (green arrow). This imbalance in electrostatic potentials in the TMD region creates an asymmetric gating mechanism. (c) The sustained binding of glycine transitions 1β GlyR to a desensitized state, returning to a more pseudo-symmetrical conformation.

We have resolved the structures of human α1β GlyR in all its major functional states, which depicts the structural rearrangements through the gating cycle (Fig. 5). In the apo state, 5-fold pseudo-symmetry is maintained in the whole α1β GlyR, with tightly constricted ion conduction pore (Fig. 5a). When glycine binds, the ECD of all 5 subunits experienced similar rotational motion, maintaining pseudo-symmetry. ECD rotation pulls on the extracellular end of TMD, resulting in the same radial motion away from the pore in all 5 subunits (Fig. 4b). However, due to electrostatics repulsion, the pore lining M2 helix of one α1 subunit moves tangentially away from the adjacent α1 subunit near the intracellular end. Since the β subunit carries opposite electrostatic charges, it remains attracted to neighboring α1 subunits. In this way, the pore is dilated in an asymmetrical manner, representing one open state of α1β GlyR. When desensitization happens, the intracellular end of M2 helices of all subunits collapse back into a more pseudo-symmetrical conformation that is thermodynamically stable^10,11,22,40^, stopping Cl^−^ conduction (Fig. 5c).

The ECD and TMD of α1 and the β subunits contributes differently to the assembling and gating of α1β GlyR. Although the unique ECD of the β subunit dictates the 4:1 α:β stoichiometry in heteromeric GlyRs^30,38,41^, it contributes similarly to α1β GlyR gating by glycine. All 5 orthorsteric pockets experience similar conformational changes from the close to the open/desensitized states, regardless of subunit types. This leads to identical structural changes in the extracellular end of TMD for all the α1 and β subunits (Fig. 2). The TMDs, on the other hand, do not affect the assembly, but instead determines functional properties of heteromeric GlyRs and contain multiple disease-causing mutagenesis sites^13,37,39^. Mutation of one residue to remove the positive charge, R271L/Q/P, of the α1, is known to diminish Cl^−^ conduction and cause hyperekplexia in a dominant manner through unclear mechanism^7,23,25^. Recently it was shown that R271 (19’) allows conduction not through locally concentrating Cl^−^, but creating electrostatic repulsion between subunits to allow the opening of the pore^42^. This is consistent with our observation of widened α1-α1, but not α1-β subunit distances in the open state because α1 and β subunits are oppositely charged at equivalent positions (Figs. 4 and 5). We believe asymmetrical expansion of pore is the most likely mechanism for a heteromeric GlyR to open. Considering the multiple single channel conductance states reported of heteromeric GlyRs^37,43^, it is tempting to speculate that multiple open state conformations exist, and some remain to be identified.

The β subunit being tightly tethered to its neighboring α1 subunits throughout the gating cycle is compatible with its role in cellular organization of GlyRs. Post-synaptic scaffolding protein gephyrin (Fig. 5 green oval), binds to the intracellular M3-M4 loop of the β subunit and clusters GlyRs at postsynaptic membranes^44–46^. Tight β-α1 interaction ensures when β subunit experiences force originating from relative motion with respect to the tethered scaffold, GlyR responds as a rigid body without changes in the ion conduction pore geometry, which may cause aberrant conduction independent of glycine binding. Since many Cys-loop receptors are clustered in post-synaptic densities, gating mechanisms that avoids excessive crossover between higher-order assembly and neurotransmitter activation may be also relevant for other receptor members, depending on specific interaction contexts.

## Methods

### Plasmid constructs

The human glycine receptor α1 (NCBI: NP_001139512.1) and β (NCBI: NP_000815.1) sequence were amplified from cDNA clones (McDermott Center, UT Southwestern Medical center). The α1em sequence was derived by substitution of M3/M4 loop (residues R316-P381) s by GSSG peptide. For the βem construct, we used the previously described βem construct^30^. The α1em and α1 wild type sequence was subcloned into a BacMam expression vector^47^. The β wild type sequence were introduced into pLVX-IRES-ZsGreen1 vector (Clonetech) for electrophysiology. All α1em and βem mutants was generated using site-directed mutagenesis.

### Protein expression and purification

The α1em and βem constructs were transformed into DH10BacY competent cells (Geneva Biotech) to produce bacmids. The bacmids were transfected into Sf9 cells to generate baculovirus. Recombinant baculovirus titer was determined as described before^30,47^. Virus was added at a multiplicity of infection (MOI) of 2(at 3βem:1α1em ratio) to the cell cultures, at a density of 2.5×10^6^ cells/ml. To increase the expression level, 10 mM sodium butyrate was added, and culture temperature was changed to 30°C after transduction 12h. Cells were collected after induction 60h by centrifugation at 30,000 g for 20 minutes at 4°C and stored at −80 °C until further use.

Cell pellets were thawed and resuspended in a by lysis buffer (40 mM Tris pH 8.0, 50 mM NaCl, 2mM MgCl2, 1mM CaCl2, 20μg/ml Dnase, 2μg/ml leupeptin, 2μM pepstatin, 0.8μM aprotinin, 0.2 mM PMSF) rotated at 4°C for 30min under constant stirring, followed by centrifugation at 40,000g for 20min to collect cell debris. The cell debris was dounced and centrifugated at 40,000g at 4 °C for 20 min. The pellets were further homogenized and solubilized with buffer A (40 mM Tris pH 8.0, 200 mM NaCl, 2mM MgCl_2_, 1mM CaCl_2_, 20μg/ml Dnase, 2μg/ml leupeptin, 2μM pepstatin, 0.8μM aprotinin, 0.2 mM PMSF, 0.75%(w/v) DDM, 0.075%(w/v) CHS and 0.075% (w/v) Na Cholate) for 40min at 4°C. Solubilized membranes were cleared by centrifugation at 40,000g for 30 min. Afterwards, supernatant was added to PA-tag antibody (NZ-1) ^48^ resin at RT. The resin was collected by a gravity column and washed with 5CV buffer B (20 mM Tris pH 8.0, 200 mM NaCl, 2mM MgCl_2_, 1mM CaCl_2_, 0.2 mM PMSF, 0.05% (w/v) DDM (Anatrace), 0.005% (w/v) CHS (Anatrace), 0.001% (w/v) Na Cholate (Anatrace)) and 5CV buffer C (20 mM Tris pH 8.0, 200 mM NaCl, 2mM MgCl_2_, 1mM CaCl_2_, 0.06% (w/v) digitonin (Sigma-Aldrich)). Then, beads were mixed with PreScission protease (1:30 v/v) to cleave PA tag at RT for 1h. The flow through was collected, and resin were washed with 2CV buffer C. All proteins were pooled and concentrated to load onto Superose6 increase 10/300 GL column (GE Healthcare) in SEC buffer (20 mM Tris pH8.0, 200 mM NaCl, 0.06%(w/v) digitonin). Peak fractions were collected and concentrated to 6 mg/ml for grids freeze.

### Cryo-EM sample preparation, data collection and image processing

For apo α1emβem GlyR, the sample was vitrified without any ligand. For glycine-bound α1β GlyR, the sample was incubated for 1h with 2 mM glycine on ice. 1 × CMC final concentration (~ 3 mM) of Fluorinated fos-choline 8 (Anatrace) was added into sample immediately before freezing. Grids (Quantifoil R1.2/1.3 400-mesh Au holey carbon grid) were glow-discharged. An FEI Vitrobot Mark IV Vitrobot (Thermo Fisher) was employed to plunge freeze the grids after application of 3 μl sample at 4°C under 100% humidity.

Micrographs were collected using a Titan Krios microscope (Thermo Fisher) with a K3 Summit direct electron detector (Gatan) operating at 300 kV using the SerialEM data acquisition software. The GIF-Quantum energy filter was set to a slit width of 20 eV. Images were recorded in the super-resolution counting mode with the pixel size of 0.415 Å. Micrographs were dose-fractioned into 50 frames with a dose rate of 1.4 e^−^/Å/frame.

2-fold binning (0.83 Å pixel size after binning), motion correction and dose weighting of the movie frames were performed using the Motioncorr2 program^49^. CTF correction was carried out using the CTFFIND 4 program^50^. The following image processing steps were carried out in RELION 3^51^, as illustrated in Supplementary Fig. 2. Particles were initially picked using the Laplacian-of-Gaussian blobs and subjected to 2D classification to obtain good class-averages, which was then used as template for reference-based autopicking. Resulting particles were extracted with 4-fold binning for a further round of 2D classification (Supplementary Fig. 2a, i). Good 2D classes were selected and subjected to 3D classification using an initial model downloaded from EMDB database (EMD-23148)^30^. For both the apo- and glycine-bound samples, 1 out of 6 classes in 3D classification appeared with good density for the entire channel (Supplementary Fig. 2b, j). A single density blob for GFP was identified for both the apo and glycine-bound samples. A further 3D classification into 4 classes with non-binned particles (0.83 Å pixel size) without particle alignment was performed. For the apo- sample, partial signal subtraction^52^ was performed to focus on the TMD. 2 indistinguishable good classes were pooled, which resulted in a final of 29, 850 particles (Supplementary Fig. 2c). After reverting particles to un-subtracted version, CTF refinement, Bayesian polishing in RELION and non-uniform refinement^53^ in cryoSPARC^54^, an overall resolution of 3.6 Å was achieved, with local resolutions exceeding 3.0 Å in many regions (Supplementary Fig. 2d, f). For the glycine-bound sample, second 3D classification was performed using a mask excluding GFP and micelle, resulting in three good classes with distinct conformations (Supplementary Fig. 2k). After CTF refinement, Bayesian polishing in RELION and non-uniform refinement in cryoSPARC, overall resolutions of 3.6 Å (21, 676 particles), 3.9 Å (24, 487 particles) and 4.1 Å (30, 723 particles) were achieved for the open, expanded-open and desensitized states, with local resolutions exceeding 3.0 Å in many regions (Supplementary Fig. 2l, n). Resolutions were estimated by applying a soft mask around the protein densities with the Fourier Shell Correlation (FCS) 0.143 criterion. Local resolutions were calculated using Resmap^55^.

### Model building and refinement

Models of GlyR α1β heteromer were bulit by fitting the structure of *Rattus norvegicus* α1β homomer glycine-bound state (PDB ID: 7mly) ^56^ into the Cryo-EM density maps of GlyR α1β heteromer using Chimera^57^ and Coot^58^. The atomic model was manually adjusted in Coot. The final models were refined with real-space refinement module and validated with comprehensive validation module in PHENIX package^59,60^. Fourier shell correlation (FSC) curves were calculated between refined atomic model and the work/free half maps as well as the full map to assess the correlation between the model and density map (Supplementary Fig. 2e and m). Statistics of cryo-EM data processing and model refinement are listed in Table S1. Pore radii were calculated using the HOLE program^61^. Figures were prepared in UCSF Chimera^57^, ChimeraX^62^, and PyMOL^63^.

The final model of apo α1β GlyR contained the α1 and β subunit amino acids except the following: α1 subunit of chain A and chain C (total 367aa, 345aa built, 22aa not built) A1-P7,L314-L315, GSSG linker, E382 and V421- Q429; α1 subunit of chain B (total 367aa, 342aa built, 25aa not built) A1-P7, H311-L315, GSSG linker, E382 and V421- Q429; α1 subunit of chain D (total 367aa, 342aa built, 25aa not built) A1-M8, K312-L315, GSSG linker, E382-E383 and V421- Q429; β subunit (total 444aa, 338aa built, 106aa not built) K1-N32, GSSAAA-EGFP-SGSGSG insertion and V378-P442.

The final model of expanded open α1β GlyR contained amino acids except the following: α1 subunit of chain A (total 367aa, 341aa built, 26aa not built) A1-P7,K312-L315, GSSG linker, E382-E383 and V421-Q429; α1 subunit of chain B (total 367aa, 339aa built, 28aa not built) A1-P7, R309-L315,GSSG linker, E382-E383 and R422- Q429; α1 subunit of chain C (total 367aa, 337aa built, 30aa not built) A1-M8, H311-L315, GSSG linker, E382-K385 and V421- Q429; α1 subunit of chain D (total 367aa, 340aa built , 27aa not built) A1-P7, H311-L315,GSSG linker,E382-E383 and V421-Q429; The model of β subunit for expanded open is the same as apo.

The final model of open α1β GlyR contained amino acids except the following: α1 subunit of chain A (total 367aa, 341aa built, 26aa not built) A1-P7,K312-L315, GSSG linker, E382-E383 and V421- Q429; α1 subunit of chain B (total 367aa, 340aa built, 27aa not built) A1-P7, R309-L315, GSSG linker, E382-R385 and V421- Q429; α1 subunit of chain C (total 367aa, 336aa built, 31aa not built) A1-M8, H311-L315, GSSG linker, E382-K386 and V421- Q429; α1 subunit of chain D (total 367aa, 340aa built, 27aa not built) A1-P7, H311-L315, GSSG linker, E382-E383 and V421- Q429; The model of β subunit for open is the same as apo.

The final model of desensitized state contained the α1 and β subunit amino acids except the following: α1 subunit of chain A (total 367aa, 335aa built, 32aa not built) A1-P7,Q310-L315, GSSG linker, E382-L387 and V421- Q429; α1 subunit of chain B (total 367aa, 336aa built, 29aa not built) A1-P7, Q310-L315,GSSG linker, E382-M384 and V421- Q429; α1 subunit of chain C (total 367aa, 337aa built, 30aa not built) A1-P7, H311-L315, GSSG linker, E382-K386 and V421-Q429; α1 subunit of chain D (total 367aa, 340aa built, 27aa not built) A1-P7, K312-L315, GSSG linker, E382-M384 and V421- Q429; The model of β subunit for desensitized is the same as apo.

### Fluorescence-Detection Size-Exclusion Chromatography (FSEC) expression assay

In the FSEC assay, fluorescence was detected using the RF-20Axs fluorescence detector for HPLC (Shimadzu, Japan) (for EGFP, excitation: 480 nm, emission: 512 nm) as EGFP was fused into βem construct. Using 2μl of Lipofectamine 3000 (Thermo Fisher Scientific, US), 1ug of plasmid (at 1α1:3β ratio) was transfected into HEK293T cells for each well of 12 well plate. Cells were incubated in a CO_2_ incubator (37 °C, 8% CO2) for 48 h after transfection and solubilized with 50μl buffer B for 1 h. After centrifugation (40,000 × g, 30 min), 50 μl of the sample was applied to a Superose 6 Increase 10/300 GL column (GE Healthcare) equilibrated with buffer D (20 mM Tris pH 8.0, 200 mM NaCl,0.025%DDM) for the FSEC assay.

## Whole Cell Patch Clamp

The glycine EC50 values were determined on α1β GlyRs expressed in HEK293T cells. Plasmids were transiently transfected using Lipofectamine 3000 reagent (Invitrogen). Total 0.8μg of DNA was transfected at 1α1:3β ratios for each 35 mm dish. Whole-cell recordings were made after 17–24h transfected at room temperature. GFP fluorescence was used to identify the cells expressing the heteromeric α1β GlyRs. The bath solution contained (in mM): 10 HEPES pH 7.4, 10 KCl, 125 NaCl, 2 MgCl_2_, 1 CaCl_2_ and 10 glucoses. The pipette solution contained (in mM): 10 HEPES pH 7.4, 150 KCl, 5 NaCl, 2 MgCl_2_, 1 CaCl_2_ and 5 EGTA. The resistance of borosilicate glass pipettes between 2~7 MΩ. For data acquisition, voltage held at −50 mV and a Digidata 1550B digitizer (Molecular Devices) was connected to an Axopatch 200B amplifier (Molecular Devices). Analog signals were filtered at 1 kHz and subsequently sampled at 20 kHz and stored on a computer running pClamp 10.5 software. Data analysis was performed by Origin software (Origin Lab). Hill1 equation was used to fit the dose-response data and derive the EC_50_ (*k*) and Hill coefficient (*n*). For glycine activation, we used $I=I_{0}+\left(I_{\max}-I_{0}\right)\frac{x^{n}}{k^{n}+x^{n}}$, where *I* is current, *I*_*0*_ is the basal current (accounting mostly for leak, very close to 0), *I*_*max*_ is the maximum current and *x* is glycine concentration. All start point is fixed at 0 during fit. Measurements were from 4–11 cells, average and S.E.M. values were calculated for each data point.

## Data Availability

The coordinates and density maps for the cryo-em data have been deposited in the Electern Microscopy Data bank under accession codes EMDB-27553 (apo state), EMDB-27552 (expanded open state), EMDB-27555 (open state), MDB-27554 (desensitized state). The coordinates have been deposited in the Protein Data Bank under accession codes 8DN3 (apo state),8DN2 (expanded open state),8DN5 (open state),8DN4(desensitized state).
